# Synthesis and characterization of porous structures of rutile TiO_2_ /Na_0.8_Ti_4_O_8_/Na_2_Ti_6_O_13_ for biomedical applications

**DOI:** 10.1016/j.mex.2019.04.002

**Published:** 2019-04-29

**Authors:** Diego Fernando Triviño-Bolaños, Rubén Jesús Camargo-Amado

**Affiliations:** Escuela de Ingeniería Química, Universidad del Valle, Ciudad Universitaria Meléndez, A. A. 25360 Cali, Colombia

**Keywords:** Synthesis and characterization of porous structures of rutile TiO_2_/Na_0.8_Ti_4_O_8_/Na_2_Ti_6_O_13_ for biomedical applications, Sodium-titanate, Porosity, Crystalline phase

## Abstract

This method involves the use of molding, pressing and sintering techniques applied to different powder mixtures of TiO_2_ with sodium bicarbonate NaHCO_3_ (15 wt% and 30 wt% NaHCO_3_), to obtain porous structures of rutile TiO_2_/Na_0.8_Ti_4_O_8_/Na_2_Ti_6_O_13_ and Na_0.8_Ti_4_O_8_/Na_2_Ti_6_O_13_ for possible biomedical implant applications. The method validation includes X-ray diffraction patterns (XRD) analysis refined by the Rietveld method using X'Pert HighScore Plus. The surface morphology was observed by using a scanning electron microscopy (SEM) equipped with an energy dispersive spectrometer (EDS), and, finally, a Chinese hamster ovary (CHO) cell line was cultured with the porous structures to determine the effect of material composition on the cellular response using a LDH cytotoxicity assay.

• The method does not require the use of toxic solvents to remove residues.
• The porous structure formed is composed mainly of crystalline phases Na_2_Ti_6_O_13_/TiO_2_ reported as biocompatible.
• It did not need complicated solid-liquid separation processes.

## Graphical abstract

**Figure:**
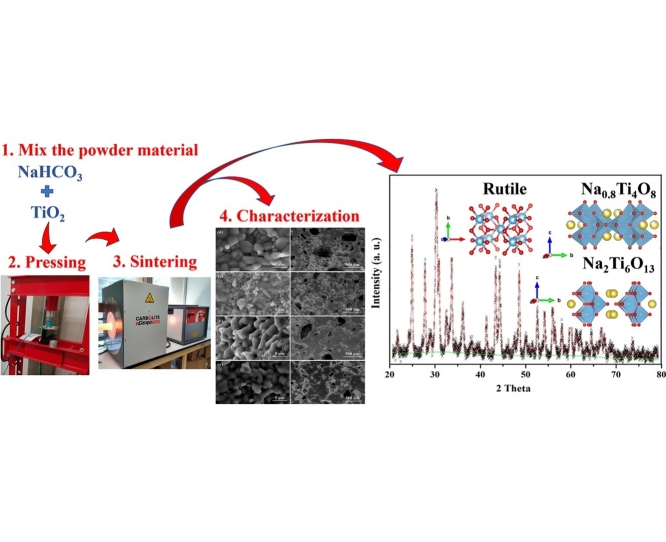

**Specifications Table**

**Table:** 

Subject area:	Materials Science
More specific subject area:	Nanostructured materials
Method name:	Synthesis and characterization of porous structures of rutile TiO_2_/Na_0.8_Ti_4_O_8_/Na_2_Ti_6_O_13_ for biomedical applications.
Name and reference of original method:	The most commonly used methods to promote porosity are: slurry foaming, salt leaching, phase separation and lyophilization. Hydrothermal treatment of TiO_2_ with NaOH is the method commonly used to obtain sodium titanates.
Resource availability:	N/A

## Method details

### Overview

In the tissue-engineering field, porous scaffolds must be biocompatible and mechanically stable. Different synthesis methods have been investigated to obtain porous materials for biomedical applications. Among the types of materials used are polymers, such as hydrogels and thermoplastics [1,2], and some types of metals, such as stainless steel [3] and gold [4]. These materials may have limitations due to its composition and synthesis method, including the lack of sufficient biocompatibility with the host, low mechanical stability [5] and, in the case of some metals, insufficient chemical stability in vivo due to the release of toxic ions [6].

Many researchers have studied different synthesis methods such as slurry foaming [7], salt leaching [8], phase separation and lyophilization [9] to promote porosity, but removing undesirable substances generally involve the use of toxic solvents [10,11], which causes cell death when they are not completely eliminated during the process. In this context, ceramic structures such as TiO_2_ and sodium titanate have good biocompatibility and chemical stability [[12], [13], [14], [15]].

Regarding to the sodium titanate preparation routes, hydrothermal treatment of TiO_2_ with NaOH is the commonly used method, however, this method requires a synthesis time of up to 24 h [16,17], and its result is a precipitation that must be separated by centrifugation and washed with abundant water and ethanol to finally obtain titanate powder [17]. In general, there are still some technological problems involved in the preparation of biocompatible porous structures, requiring several high-cost synthesis methods.

**Fig. 1:**
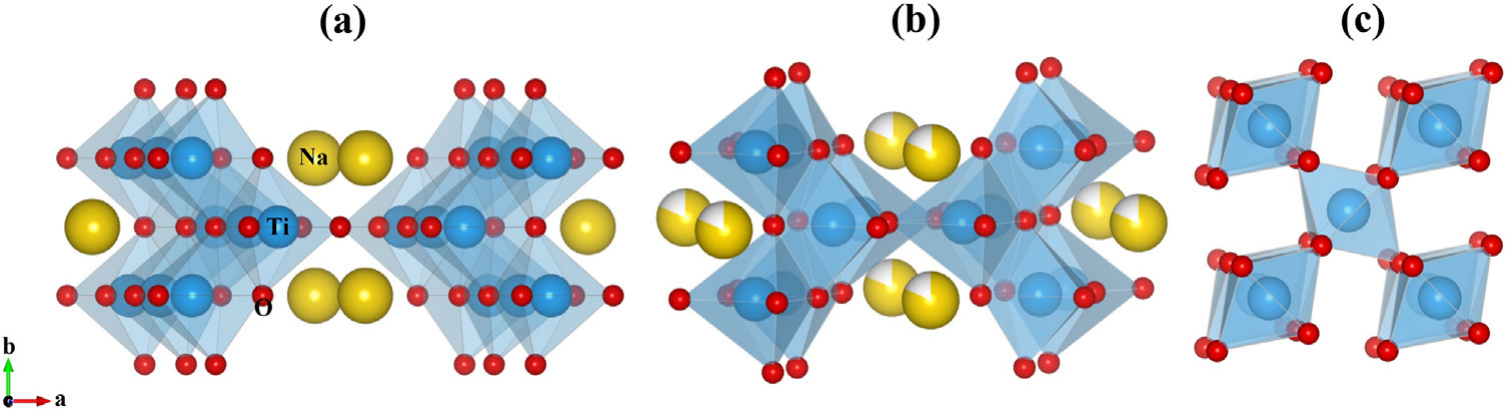
Crystal structure of (a) Na_2_Ti_6_O_13_, (b) Na_0.8_Ti_4_O_8_ and (c) TiO_2_ rutile.

This paper proposes the following method: By applying molding, pressing and sintering techniques in mixtures of TiO_2_/NaHCO_3_ powder materials, it was possible to obtain porous rutile TiO_2_/Na_0.8_Ti_4_O_8_/Na_2_Ti_6_O_13_ composites (Fig. 1) of the required size and shape, with minimal variations and adequate mechanical properties. Unlike traditional methods, this method does not require the use of toxic solvents to remove residues, because the porous structure formed is mainly composed of crystalline phases of Na_2_Ti_6_O_13_/TiO_2_ reported as biocompatible [14,18] with adequate chemical stability [[12], [13], [14], [15]]. Another advantage of this method was the porosity and roughness control by varying the amount of NaHCO_3_ and the sintering time [19].

At 230 °C the Na_2_CO_3_ formation (Eq. (1)) responds to a reaction of NaHCO_3_ at 230 °C [20].

(1) $$2\text{N}\text{a}\text{H}\text{C}{\text{O}}_{3}\left(\text{s}\right)\overset{230\;{}^{\circ}\text{C}}{\to }{\text{N}\text{a}}_{2}\text{C}{\text{O}}_{3}(\text{s})+\text{C}{\text{O}}_{2}\left(\text{g}\right)+{\text{H}}_{2}\text{O}(\text{g})$$

Then the solid-state reaction between Na_2_CO_3_ and TiO_2_ (anatase or rutile phase) takes place at temperatures around of 800 °C [[21], [22], [23]]. Na_2_CO_3_ can also be transformed (Eq. (2)) into Na_2_O [24]:

(2) $${\text{N}\text{a}}_{2}{\text{C}\text{O}}_{3}\to {\text{N}\text{a}}_{2}\text{O}+{\text{C}\text{O}}_{2}$$

The release of gases during the heating treatment can promote voids in the structure. Depending on the molar concentration of Na_2_O and TiO_2_, both can react in the solid state to form different types of sodium titanates according to the binary Na_2_O-TiO_2_ phase diagram [25]. For a high molar content of TiO_2_ (>85 mol%), the main phases formed according to the phase diagram are TiO_2_ rutile and Na_2_Ti_6_O_13_ [22]. Based on the literature, the formation of Na_2_Ti_6_O_13_ occurs via the decomposition of Na_2_Ti_3_O_7_ at temperatures from 400 °C to 1100 °C [15].

In this paper, porous structures of rutile TiO_2_/Na_0.8_Ti_4_O_8_/Na_2_Ti_6_O_13_ and Na_0.8_Ti_4_O_8_/Na_2_Ti_6_O_13_ have been synthesized by molding, pressing and sintering different mixtures of TiO_2_/NaHCO_3_ powder materials. We study the effects of the sintering time and the weight ratio of TiO_2_/NaHCO_3_ on the porosity, and structure proportion of rutile TiO_2_ wt%/Na_0.8_Ti_4_O_8_ wt%/Na_2_Ti_6_O_13_ wt% and Na_0.8_Ti_4_O_8_ wt%/Na_2_Ti_6_O_13_ wt% crystalline phases. Likewise, the biological properties of the materials studied are evaluated in-vitro through cytotoxicity tests with the CHO cell line.

## Experimental

### Sol-gel synthesis

**Fig. 2:**
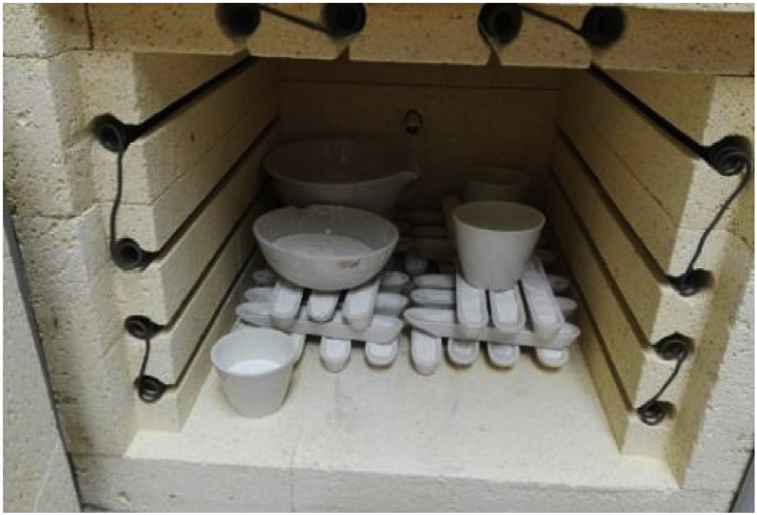
TiO_2_ powder samples sintered at 900 °C.

6 g of TiO_2_ were prepared by the sol-gel method. In a typical synthesis, 21 mL of titanium(IV) n-butoxide, 99% (TBT-ACROS) were mixed with 76 mL of absolute ethanol (Aldrich 99.8%) for 10 min under magnetic stirring. Then, 2 mL of deionized distilled water were added at the rate of 1 mL min^−1^ to promote the gelation of the components. The solution was heated at 100 °C for 10 days. Subsequently, the TiO_2_ powder was placed inside a muffle furnace (see Fig. 2), heated at the rate of 3.5 °C min^−1^, sintered at 900 °C for 3 h, and cooled down to 25 °C at the rate of 5 °C min^−1^.

### Mixing and pressing

**Fig. 3:**
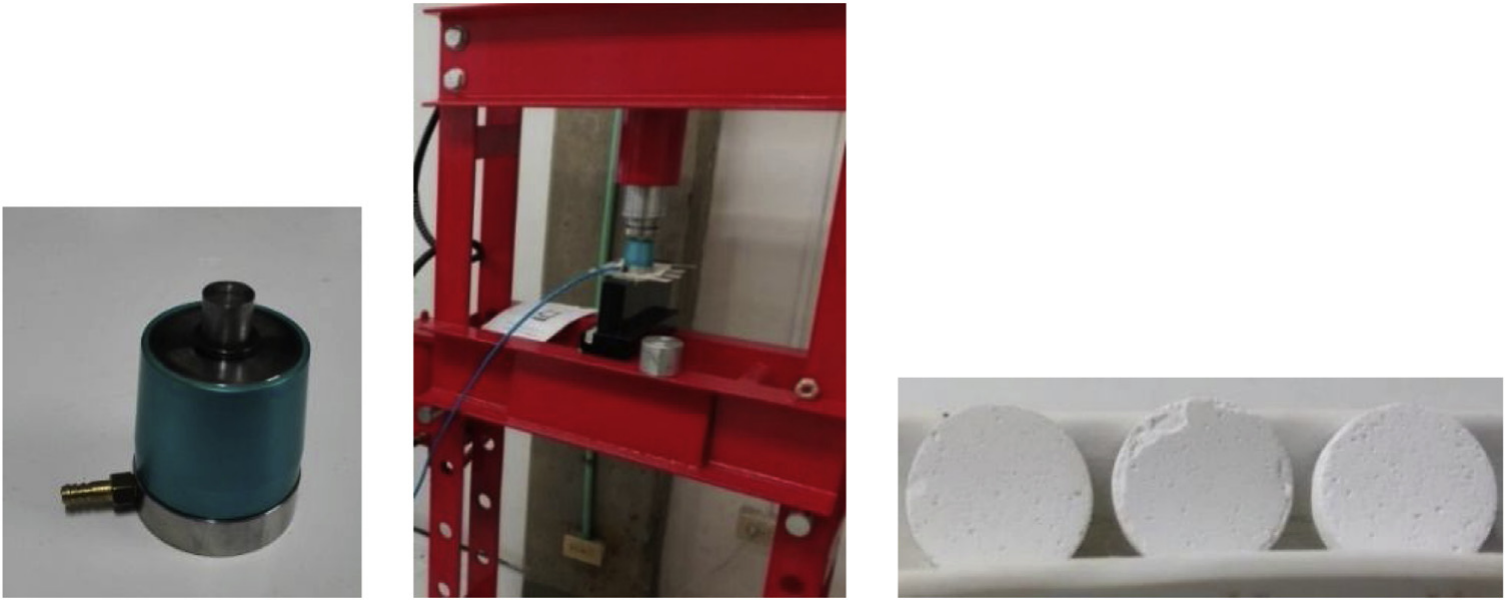
Mold and uniaxial press used to obtain the green body.

The TiO_2_ and NaHCO_3_ powders were ball-milled using a Spex Mixer/Mill for 10 min and 2 min respectively. Subsequently, both materials (TiO_2_ with 15 wt% NaHCO_3_; TiO_2_ with 30 wt% NaHCO_3_) were mixed manually. Then, the mixture was poured into a steel mold (13 mm in diameter and approximately 3 mm in height) and pressed uniaxially (35 Kg cm^−2^) for 5 min using a hydraulic press to form green compacts, as can be seen in Fig. 3.

### Sintering

**Fig. 4:**
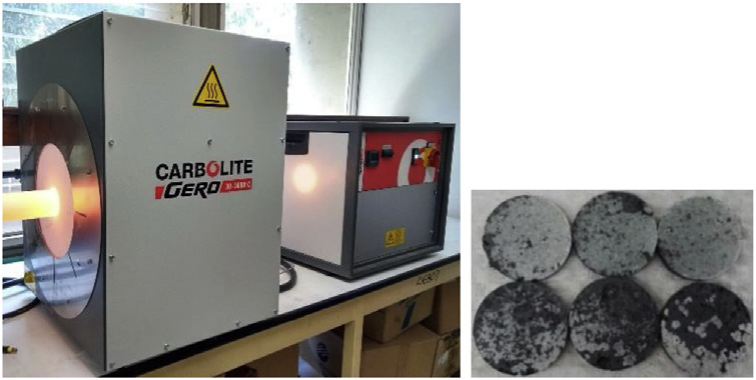
Sintered disc samples 3 mm thick and 12 mm–13 mm in diameter.

After drying at 45 °C for 2 h, the green compact discs were sintered in a tube furnace (Carbolite Gero HTRH) at 1200 °C for 4 h and 8 h in an inert atmosphere (Ar) with a flow rate of 1 L min^−1^ (Fig. 4). The heating rate was 3.5 °C min^−1^ and then cooled to 25 °C at a rate of 5 °C min^−1^. For all tests, the discs of rutile TiO_2_/Na_0.8_Ti_4_O_8_/Na_2_Ti_6_O_13_ were polished with 800 and 1200 SiC paper for 2 min each side, and ultrasonically rinsed in acetone for 20 min to remove impurities.

### Surface characterization

The characterization of the prepared materials was carried out to validate the formation of rutile TiO_2_/Na_0.8_Ti_4_O_8_/Na_2_Ti_6_O_13_ and Na_0.8_Ti_4_O_8_/Na_2_Ti_6_O_13_ porous structures. XRD patterns were obtained using a Rigaku DMAX 2100 diffractometer; *CuK*_α_ radiation (1.54056 Å and 1.54439 Å) was used as an X-ray source in the 2θ range of 20-80°, with a step size of 0.02°.

### Identification and quantification of crystalline phases using X'Pert HighScore Plus

Moving forward, the samples prepared under different conditions will be referred to as **T1**: TiO_2_ with 15 wt.% of NaHCO_3_ at 1200 °C for 4 h; **T2**: TiO_2_ with 15 wt.% of NaHCO_3_ at 1200 °C for 8 h, **T3**: TiO_2_ with 30% NaHCO_3_ at 1200 °C for 4 h; and **T4**: TiO_2_ with 30 wt.% NaHCO_3_ at 1200 °C for 8 h.

For the XRD patterns of the materials obtained at conditions T1, T2, T3 and T4, the following methodology was used to identify and quantify three crystalline phases in the materials using the PANalytical X'Pert HighScore Plus software. For the T1 sample, the following analysis procedure was used: Treatment→Search peaks:

Search programming: “Analysis”→ “Search & Match”→ “Execute Search & Match”:

Search programming: “Restriction”→ “Edit Restriction Sets”

Search programming: Select possible elements present in the sample(Na, Ti and O):

Select the compounds that most fit the diffractogram

**Fig. 5:**
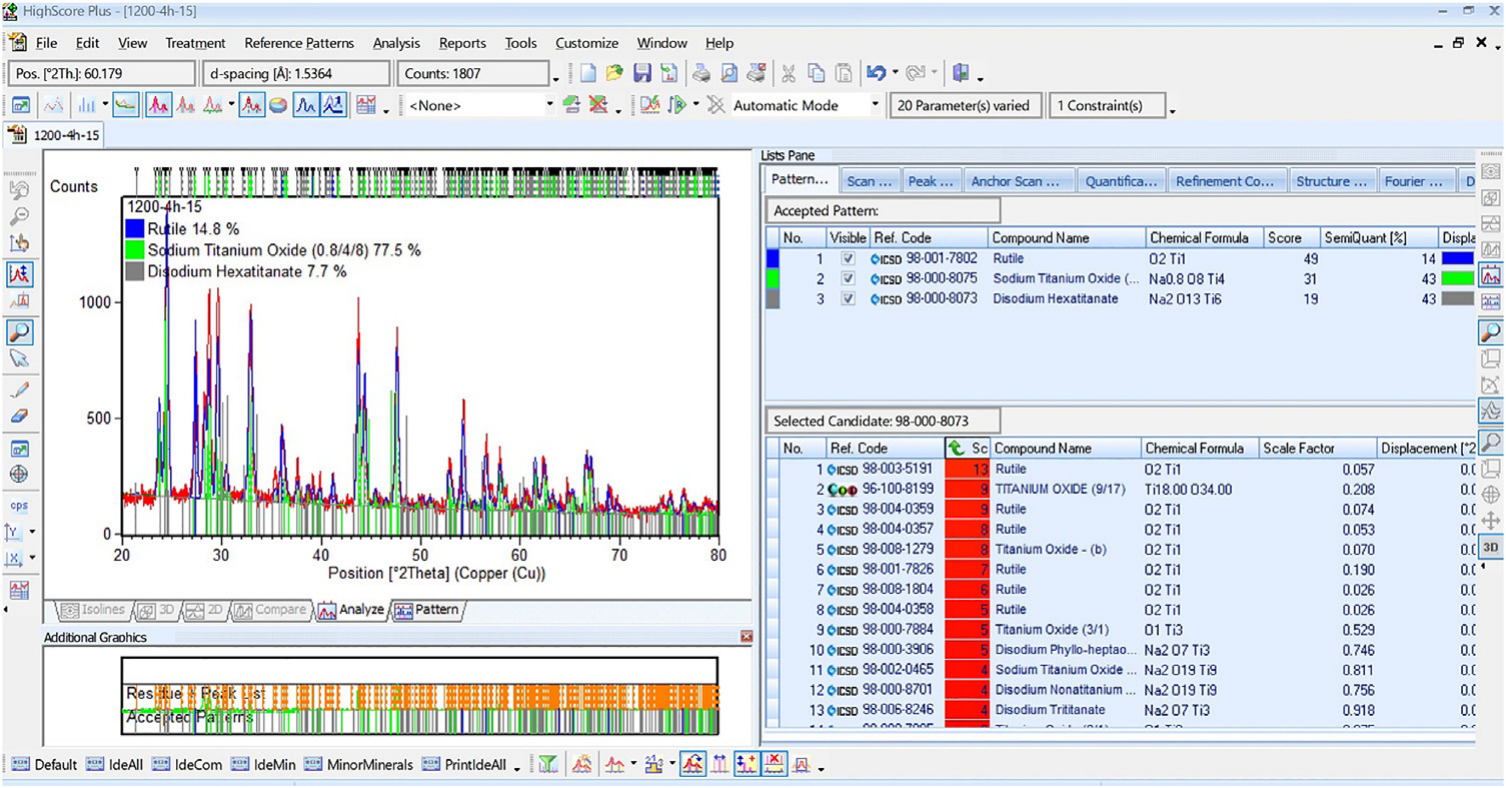
Refinement results by the Rietveld method using X'Pert HighScore Plus software for sample T1.

It is also possible to refine the diffractogram by using the Rietveld method to identify the major crystalline phase proportion (wt%) (Fig. 5):

“Analysis” → “Rietveld” → “Star Rietveld Refinement”

### Determination of crystallographic orientation

For all samples, it was possible to identify the preferential orientation of the more intense peaks of the crystalline phases. Here are the steps taken: Right click over the refined diffractogram → Label Peaks→Select: n most intense Peaks (for this diffractogram, it is calibrated up to 9). In Labels select: Phase Title and the boxes.

### Morphological analysis

The morphology of the materials was characterized using a Phillips ESEM XL30 scanning electron microscope (SEM), operating at 20 kV. The associated energy-dispersive spectrometer (EDS) provided quantitative information about surface elemental composition. The images were obtained at magnifications of 65X and 5000 × . The pore sizes and surface porosities of the materials were quantified by digital image analysis using ImageJ®

### Cytotoxicity test

Cytotoxicity in-vitro tests of 24 h and 48 h were performed using the cytotoxicity Kit LDH [26], which quantifies death and cell lysis based on the measurement of lactate dehydrogenase activity (LDH), released from the cytosol of damaged cells. The biological procedures were carried out in a pre-sterilized laminar flow hood. The working solutions and Kit LDH controls were prepared and applied according to protocol. The LDH release was measured at 24 and 48 h using an Elisa microplate reader (iMark Bio-Rad), with a working length of 492 nm and a reference length of 655 nm.

## Results and discussion

The diffraction peaks of the rutile TiO_2_, disodium hexatitanate (Na_2_Ti_6_O_13_) and sodium titanate (Na_0.8_Ti_4_O_8_) crystalline phases were consistent with the standard patterns (PDF# 98-001-7802, PDF# 98-000-8073 and PDF # 98-000-8075 respectively) and showed tetragonal-monoclinic-monoclinic structures with spatial groups of P 42/m n m, C 2/m, and C 2/m, respectively.

**Fig. 6:**
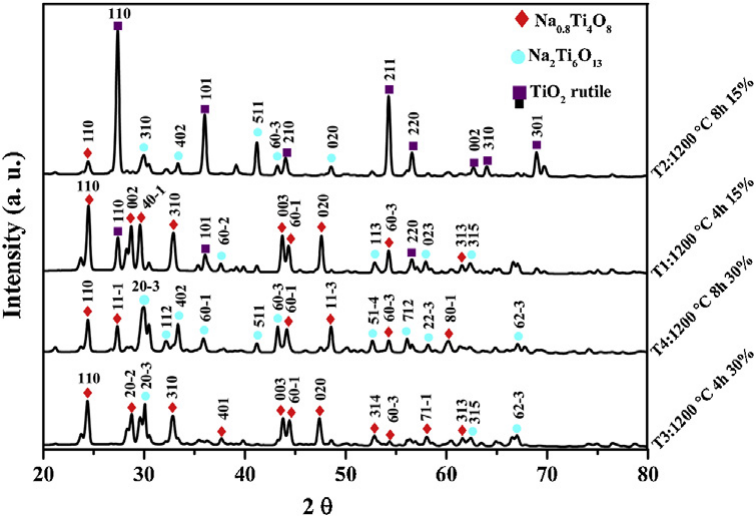
Diffraction peaks of the TiO_2_ rutile, Na_2_Ti_6_O_13_ and Na_0.8_Ti_4_O_8_ under T1-T4 condition.

By organizing the data and graphing it in OriginLab® (Fig. 6) for T1, the diffraction peaks at the Bragg angles of 27.4°, 36.1° and 56.5°, are attributed to TiO_2_ rutile (110), (101) and (220) crystalline plane respectively. Diffraction peaks (110), (002), (40-1), (310), (003), (60-1), (020), (60-3) and (313) Na_0.8_Ti_4_O_8_ crystalline planes appeared at 2 θ = 24.5°, 28.7°, 29.7° 32.8°, 43.6°, 44.3° 47.6°, 54.3° and 61.4° respectively.

For T2, the diffraction peaks (110), (101), (210), (211), (220), (002), (310) and (301) TiO_2_ rutile planes showed a preferred orientation at 2 θ = 27.3°, 36°, 43.9°, 54.2°, 56.4°, 62.6°, 63.8° and 68.8° respectively. The peaks at around 29.7°, 33.3°, 41.1°, 43.1° and 48.5° are ascribed to Na_2_Ti_6_O_13_ (310), (402), (511), (603) and (020) planes, respectively. The diffraction peak at around 24.4° is attributed to Na_0.8_Ti_4_O_8_ (110) crystalline plane.The XRD pattern of T3 exhibited diffraction peaks at around 24.3°, 28.7°, 32.9°, 37.5°, 43.7°, 44.4°, 47.3°, 52.8°, 54.4°, 58° and 61.4° attributed to Na_0.8_Ti_4_O_8_ (110), (20-2), (310), (401), (003), (60-1), (020), (314), (60-3), (71-1) and (313) crystalline planes respectively. The diffraction peak at around 30.1°, 62.4°, and 66.9° are attributed to Na_2_Ti_6_O_13_ (20-3), (315) and (62-3) crystalline planes.

In the case of T4, Na_2_Ti_6_O_13_ showed that its diffraction peaks could be indexed to the (20-3) (30°), (112) (32.2°), (402) (33.4°), (60-1) (35.8°), (511) (41.8°), (60-3) (43.2°), (51-4) (52.6°), (712) (56.1°), (22-3) (58.2°) and (62-3) (67°) planes. Finally, the diffraction peaks at the Bragg angles of 24.3°, 27.2°, 44.3°, 48.5°, 54.3° and 60.2°, are attributed to Na_0.8_Ti_4_O_8_ (110), (11-1), (60-1), (11-3), (60-3), and (80-1) crystalline planes respectively.

### Effects of sintering time and NaHCO_3_ percentage on the crystalline phases

At T1, the results showed three crystalline phases, in different proportions: 14.8 wt% of rutile TiO_2_, 7.7 wt% of Na_2_Ti_6_O_13_ and 77.5 wt% of Na_0.8_Ti_4_O_8_, as can be seen in Table 1. The majority phase was sodium titanate or titanium bronze Na_0.8_Ti_4_O_8_. According to previous research [27], this phase is formed from the reduction of Na_2_Ti_3_O_7_ in inert gas atmosphere (hydrogen) at 950 °C for two days. As previously mentioned, the formation of Na_2_Ti_6_O_13_ also requires the reaction of Na_2_Ti_3_O_7_ at temperatures around 400 °C to 1100 °C. However, the Na_2_Ti_3_O_7_ phase was not observed in any of the samples analyzed, which suggests that this phase (under reducing atmosphere), can be transformed into Na_0.8_Ti_4_O_8_ or Na_2_Ti_6_O_13._

**Table 1:** Rietveld refinement values from XRD patterns of T1, T2, T3 and T4 samples.

Sample	Phase fraction (wt%)		
	Na_2_Ti_6_O_13_	Na_0.8_Ti_4_O_8_	Rutile
T1:1200 °C- 4h-15%	7.7	77.5	14.8
T2:1200 °C-8h-15%	27.7	1.5	70.8
T3:1200 °C-4h-30%	27.6	72.4	–
T4:1200 °C-8h-30%	84.1	15.9	–

When comparing the T1 with T2 results, it is observed that in T2, higher concentration of the rutile TiO_2_ and Na_2_Ti_6_O_13_ phases were obtained, while the percentage of Na_0.8_Ti_4_O_8_ was lower. The main difference between T1 and T2 is the increase of the sintering time from 4 h to 8 h. As the sintering time increases, it is possible that more Na_2_Ti_6_O_13_ is converted into rutile TiO_2_ as it occurs at T2 condition [15]. The presence of rutile TiO_2_ and Na_2_Ti_6_O_13_ in the T1 and T2 samples corresponds to Na_2_O-TiO_2_ phase diagram [25], due to the high mole amounts of TiO_2_ (> 85 mol% TiO_2_).

While at T3, the proportion of phases obtained was 27.6 wt% of Na_2_Ti_6_O_13_ and 72.4 wt% of Na_0.8_Ti_4_O_8_. No rutile TiO_2_ peaks were observed in the T3 and T4 samples, probably due to the high content (wt%) of NaHCO_3_. When comparing this result with the one obtained under condition T4, it was found that the proportion of Na_2_Ti_6_O_13_ was higher while the proportion of the Na_0.8_Ti_4_O_8_ phase was lower. It is possible that a sintering time of 8 h promotes higher formation of the Na_2_Ti_6_O_13_ phase. The main differences between T1-T2 and T3-T4 is the increase on the percentage (wt%) of NaHCO_3_.

### SEM images and EDS pattern

**Fig. 7:**
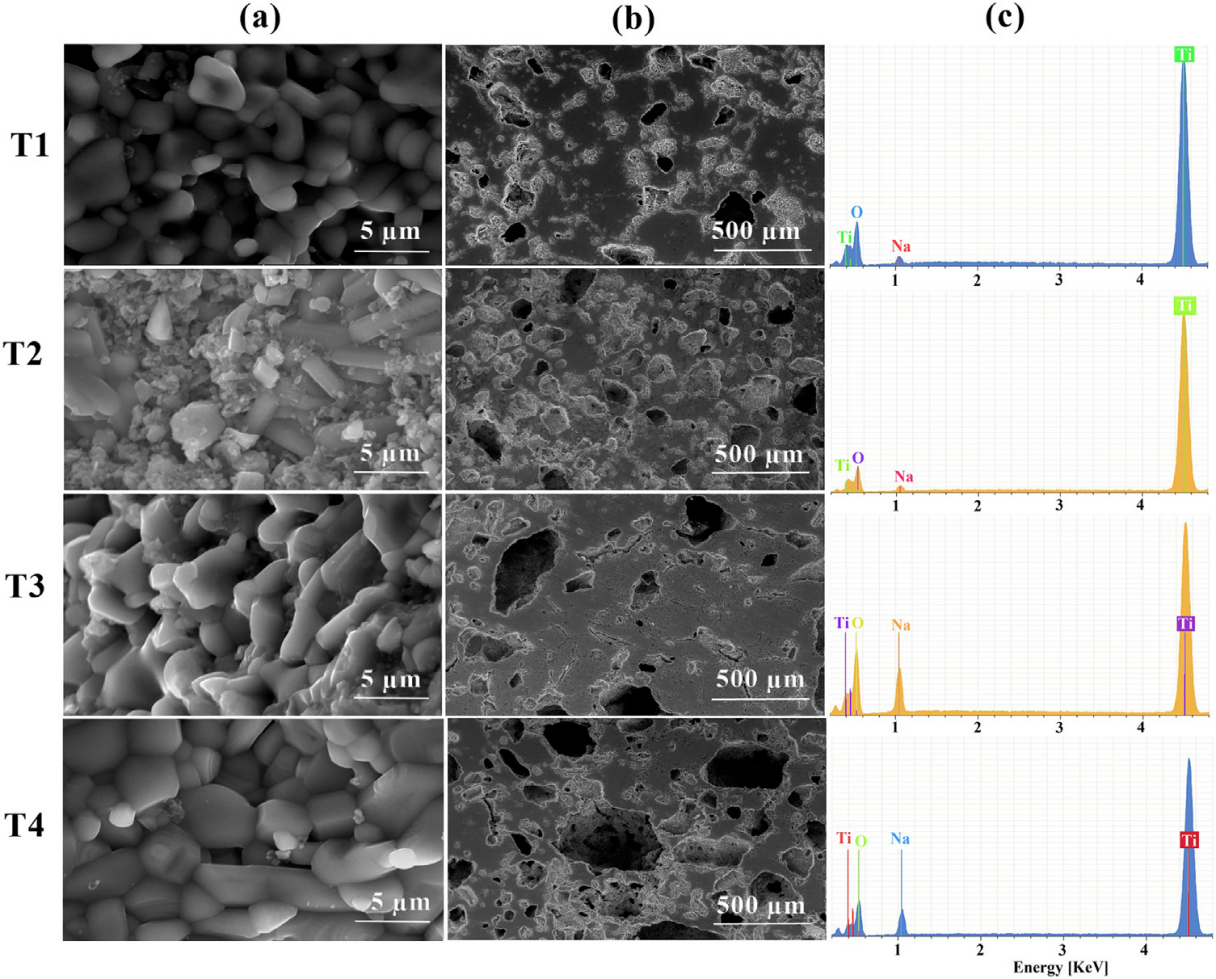
SEM images of porous structures rutile TiO_2_/Na_0.8_Ti_4_O_8_/Na_2_Ti_6_O_13_ and Na_0.8_Ti_4_O_8_/Na_2_Ti_6_O_13_ at T1, T2, T3 and T4 conditions. (a) 5000X, (b) 65X, (c) EDS analyses of the surface composition.

The scanning electron micrographs showed particle sizes with different morphologies (Fig. 7T1(a)): small spherical particles of 1 μm in diameter and semi-cubic particles of about 3 μm in size. The maximum pore size was 170.5 μm (Fig. 7T1(b)). The grain sized with polygonal structures [28] most probably due to the highest sintering time, is shown in Fig. 7T2(a). Similar results have been reported by other authors [15,22] calcining Na_2_Ti_3_O_7_ from 800 °C for several hours. Fig. 7T2(b) exhibited a maximum pore size of 180 μm.

When comparing, the T3(b)-T4(b) samples, showed higher surface porosity than the T1(b)-T2(b) samples. It is possible to observe that the porosity increased as the amount (30 wt%) of NaHCO_3_ and the sintering time (8 h) increased. In other words, the higher the porogenic percentage and the sintering time, the greater the porosity.

Likewise, it is observed that the grain growth continued with the increase of the sintering time, as can be seen in Fig. 7T2(a) and T4(a). Grain boundaries are evident in the T4(a) sample, demonstrating the good crystallinity and sintering property.

The corresponding elemental composition of the materials is shown in Fig. 7(c), and the elemental ratios as obtained from de EDS detector are presented in Table 2. EDS analysis revealed oxygen, sodium and titanium to be present on the surface.

**Table 2:** Elemental ratios obtained from EDS results.

Condition	Element	Atomic %	Weight %
T1	O	69.84	44.61
	Na	2.27	2.08
	Ti	27.89	53.31
	Totals	100	100
T2	O	63.75	37.8
	Na	2.32	1.98
	Ti	33.93	60.22
	Totals	100	100
T3	O	68.78	46.83
	Na	9.86	9.65
	Ti	21.36	43.52
	Totals	100	100
T4	O	64.42	40.62
	Na	7.91	7.17
	Ti	27.67	52.21
	Totals	100	100

Depending on the tissue application, appropriate pore sizes can vary from 5 to 500 μm in diameter. For bone tissue engineering applications and designing of macro devices to treat cellular immunodeficiency diseases, the optimal pore diameters vary from 40 to 450 μm [3,29]. In this context, the maximum pore size of the samples obtained at the different treatment conditions were calculated between 50 and 496 μm, which is a widely accepted range for use in biomedical applications.

### Cytotoxicity evaluation

The success in the preparation and characterization of any material for use in the biomedical field depends on the positive response that cells, and tissues present upon being in contact with such material once implanted.

**Fig. 8:**
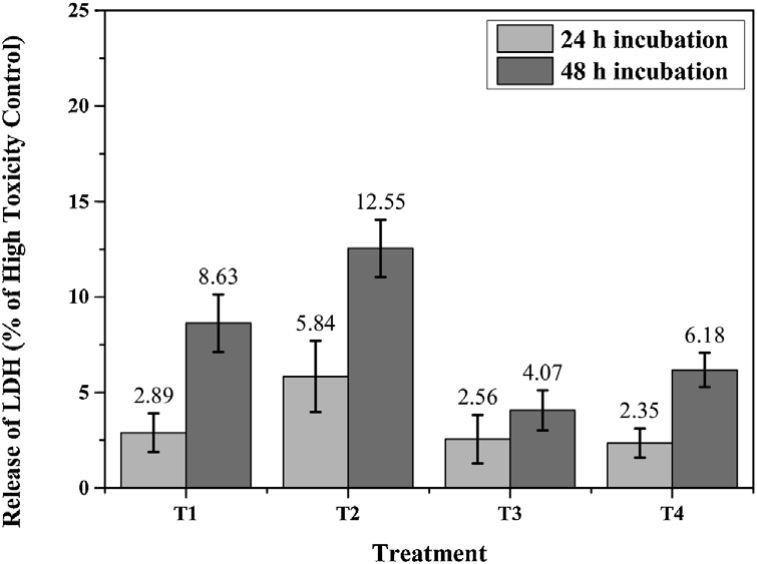
Results of LDH test after 24 h and 48 h of incubation.

The LDH release results at 24 h and 48 h from the CHO cell line showed in Fig. 8. LDH release from all materials in the presence of the cells was low compared to high toxicity control of 1% Triton-X 100

The materials (T3 and T4) with more NaHCO_3_ (30 wt%) constituted by the Na_2_Ti_6_O_13_ and Na_0.8_Ti_4_O_8_ phases caused less LDH release at 24 and 48 h than the materials (T1 and T2) constituted by rutile TiO_2_, Na_2_Ti_6_O_13_ and Na_0.8_Ti_4_O_8_ phases prepared with less NaHCO_3_. It is possible that the presence of the rutile phase in the T1 and T2 samples increased the LDH release compared to the materials constituted by Na_2_Ti_6_O_13_ and Na_0.8_Ti_4_O_8_ phases. In previous investigations, sodium titanates were prepared via hydrothermal process and sintered at temperatures between 900–1050 °C for 1 h [14]. These materials presented low LDH release in the cytotoxicity assay.

The in-vitro biocompatibility of porous barium titanate manufactured by a direct foaming technique was investigated, using an LDH concentration assay on osteoblast cells cultured for 72 h [29]. In all cases, the results showed that the materials were significantly less toxic to cells, than those cultured with 1% Triton X-100 for 45 min (high toxicity control).

It was demonstrated that the materials evaluated (TiO_2_/Na_0.8_Ti_4_O_8_/Na_2_Ti_6_O_13_ and Na_0.8_Ti_4_O_8_/Na_2_Ti_6_O_13_) in the present investigation induce low LDH release (less than 13%).

## Conclusion

Porous structures composed of the rutile TiO_2_/Na_0.8_Ti_4_O_8_/Na_2_Ti_6_O_13_ and Na_0.8_Ti_4_O_8_/Na_2_Ti_6_O_13_ crystalline phases were obtained by mixing, compacting and sintering TiO_2_ with NaHCO_3_ at different times in an inert Ar atmosphere. It can be concluded that varying these treatment conditions influences the response variables, such as porosity and structure proportion. Moreover, these properties can be controlled to produce a potential suitable material for use in the biomaterials field.

According to the results of the cytotoxicity test, the ceramic porous structures under study induce low LDH release in the presence of the CHO cell line in vitro, regardless of the treatment condition and the rutile TiO_2_/Na_2_Ti_6_O_13_/Na_0.8_Ti_4_O_8_ phase fraction, the porous ceramic materials evaluated could be used for biomedical applications. Finally, this method of synthesis could be used for future applications in the biomedical field, replacing conventional manufacturing techniques because of its porous structure and specific crystalline phases that do not affect the in-vitro cell viability.
